# Unsolicited solicitations: identifying characteristics of unsolicited emails from potentially predatory journals and the role of librarians

**DOI:** 10.5195/jmla.2022.1554

**Published:** 2022-10-01

**Authors:** Paije Wilson

**Affiliations:** 1 paije.wilson@wisc.edu, Health Sciences Librarian, University of Wisconsin-Madison, Madison, WI.

**Keywords:** Commentary, publishing, fraud, predatory journals, librarians, scholarly communications

## Abstract

Email solicitations for manuscript submissions are a common tactic employed by predatory journals to attract potential victims. Both new and established researchers alike have fallen prey to this tactic, justifying the need for librarians to provide further education and support in this area.

This commentary provides a succinct overview of predatory journals; briefly describes the problem of predatory journal email solicitations; explains the role librarians can play in their identification; and lists some red flags and tactics librarians can tell researchers to look out for, as informed by the literature and the author's analysis of 60 unsolicited journal emails she received in her own institutional inbox.

## A BRIEF OVERVIEW OF PREDATORY JOURNALS

Predatory (aka fraudulent) journals have become increasingly common in academia. In their 2015 study, Shen & Björk estimated that over 400,000 items per year were being published in predatory journals [1], and Moher et al. estimated in 2017 that at least 18,000 funded biomedical research studies had been published in predatory journals [2]. Despite their prevalence, there is no standard definition for predatory journals, a shortcoming likely attributed to their broad, and variable range of characteristics [3-6]. However, the term “predatory journal” generally refers to journals that have no quality control in their selection process; have little to no peer review, editorial, or preservation services; and exploit open access publication models for financial gain [3, 4, 6-8]. Some common characteristics of predatory journals and how to identify them have been compiled in the literature [2, 4-10].

Established and new scholars, from both developing and prestigious institutions alike, have fallen victim to predatory journals, an outcome which researchers theorize may be the product of both the “publish or perish” mentality of academia (i.e., where scholars are pressured to quickly publish research for tenure and promotion) and simple lack of guidance and training on how to identify a potentially predatory journal [2, 3, 6, 11, 12]. For researchers, the consequences of submitting manuscripts to a predatory journal are numerous and may include professional embarrassment, loss of publishing opportunities (i.e., publishing research in a predatory journal could prevent the research from being published elsewhere), a negative effect on their impact metrics (as predatory journals typically have fake or inexistent impact metrics, due to their inability to pass quality checks in reputed databases such as Web of Science Core Collection and Scopus), and loss of the money the researcher may have paid for the journal's article processing charge (APC) [4, 5, 13].

## THE PROBLEM OF PREDATORY JOURNAL EMAIL SOLICITATIONS

According to Cobey et al.'s 2018 survey of 82 researchers that had manuscripts published in predatory journals, 41% of the researchers' first encounters with the predatory journal were from receiving an unsolicited email for manuscript submission [11]. This percentage is quite significant and justifies the need to educate researchers on how to identify manuscript solicitations deriving from potentially predatory senders. This is a role librarians may take on, as professionals who commonly hold responsibilities relating to scholarly communications support. Doing so may reduce the efficacy of a formidable tactic employed by predatory journals, enabling researchers to think critically about solicitations they receive in their inboxes and alerting them to the need to further investigate a journal's legitimacy prior to submitting a manuscript.

However, though some studies have specifically focused on the recipients or characteristics of email solicitations from predatory journals [14-21], few have done granular analyses of common characteristics of unsolicited journal emails, with exceptions including Mercier et al. (2018), which examined emails sent to a recently graduated emergency physician; McKenzie et al. (2021), which examined emails sent to a surgeon; Clemons et al. (2017), which examined emails sent to a medical oncologist; and Sousa et al. (2021), which examined emails sent to an academic in the School of Dentistry [14, 16, 19, 21]. So, with such a deficiency in the literature, how can librarians and researchers identify these emails?

## CHARACTERISTICS TO LOOK OUT FOR IN UNSOLICITED JOURNAL EMAILS

In spite of the sparse literature on identifying predatory email solicitations, there are some red flags common to predatory journals that librarians and researchers can apply when scrutinizing unsolicited manuscript solicitations, with the addition of the red flags identified in the few studies examining the content of potentially predatory solicitations. Cumulatively, these red flags include:

- **Spamming**: Predatory journals are known to aggressively “spam” potential victims with unsolicited manuscript solicitations [3, 4, 6-8, 10, 11, 14-16, 19]. Researchers should be critical of journals that send unsolicited emails soliciting manuscripts, and journals that send unprompted follow-ups to said solicitations.
- **Short deadlines for manuscript submission**: Predatory journals frequently attempt to instill a sense of urgency in researchers to submit their work [14, 16, 19]. Researchers should be cautious of emails that have a particularly short deadline for article submission.
- **Non-personalized or erroneous salutations**: Predatory journals may lack personalized salutations in their email solicitations, as they are attempting to target a broad range of researchers [14, 22]. Researchers should be cautious of email solicitations that lack personalized salutations (e.g., “Dear Researcher”), or that have errors in their salutations (e.g., addressing a researcher by the honorific “Dr” when they don't have a PhD).
- **Scope that doesn't match the researchers' field**: Predatory journals have the tendency to be broad in scope, with the intention of attracting researchers from most any discipline [2, 7, 8, 10, 15, 16, 19]. Researchers should be suspicious of email solicitations from journals that have an exceptionally broad scope, and/or that have scopes that do not align with their or their past publications' field of study.
- **Additional red flags**: Other well documented characteristics of predatory journals include grammar/spelling errors, email addresses that don't reflect the journal's name, flattery, allowance for email submissions (i.e., as opposed to a formal manuscript submission system), persuasive language (e.g., listing why an author should publish with the journal), fake impact factors, a nonprofessional email address (i.e., a Yahoo or Gmail email address), and offering discounts for publication [2, 4, 6-8, 10, 19, 21, 22]. Researchers should take caution of email solicitations that have any of the above characteristics.

**Figure 1:**
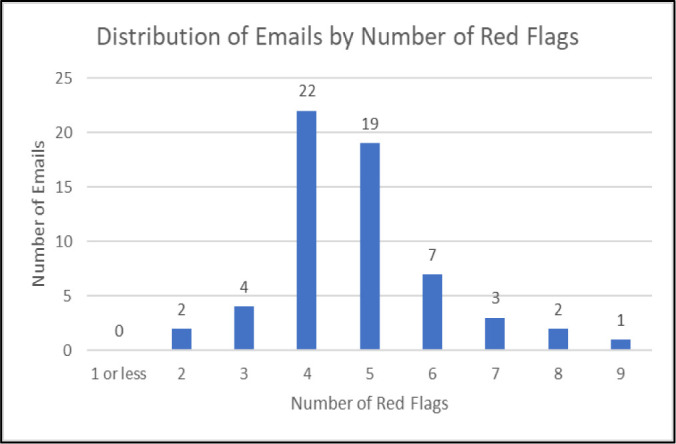
Distribution of 60 unsolicited journal emails received between July 20, 2021, and January 31, 2022, by number of red flags observed in their contents. For example, 2 emails had only 2 red flags, 4 emails 3 red flags, 22 emails 4 red flags, etc.

Additionally, librarians can take an active role in noting the red flags that appear in unsolicited journal emails received within their own inboxes to better inform their support for researchers who may receive similar emails. Below, in Table 1, the author includes her own observations of red flags in her exploratory analysis of 60 unsolicited journal emails she had received between the dates of July 20, 2021 (being the date her first manuscript was published) and January 31, 2022, in her institutional inbox. Figure 1 displays a distribution of the emails by the number of red flags observed in their content. Collecting data such as these can help librarians to get an impression of the existence, prevalence, and number of red flags that may be present in email solicitations from potentially predatory journals, all being vital insights they can use to inform their instruction on this topic.

**Table 1:** Prevalence of red flags from 60 unsolicited journal emails received between July 20, 2021, and January 31, 2022. Characteristics are in the order of most to least prevalent.

Red Flags	Number of Emails (n=60)
Grammar/spelling errors	60 (100%)
Email address did not reflect the journal's name	33 (55%)
Scope did not match author's field	29 (48%)
Incorrect honorific in salutation (e.g., Dr., as author does not have a PhD)	28 (47%)
Flattery	27 (45%)
Allow for email submissions	24 (40%)
Deadline mentioned	21 (35%)
Persuasive language	18 (30%)
No name in salutation (generic, e.g., “Dear researcher”)	11 (18%)
Scope matched author's field because extremely broad	10 (17%)
Follow ups	10 (17%)
Yahoo or Gmail email address	6 (10%)
Discount mentioned	5 (8%)
Incorrect name in salutation	4 (7%)
Fake impact metric mentioned	1 (2%)

* Of note, all but 6 of the emails in the author's sample were confirmed to be derived from journal titles and/or publishers included in Beall's List of Potential Predatory Journals and Publishers (i.e., 54 of the 60 emails were from journals and/or publishers on beallslist.net) [23], with the remaining 6 emails having at least 4 or more of the red flags identified in the table. While these factors do not necessarily mean all of the emails in the sample were from predatory journals, they are an indication that the journals may have been of low quality or predatory in nature.

For example, based on the findings from the author's limited sample, librarians may consider creating example emails containing 4 or 5 of the more prevalent red flags listed below, such as grammar/spelling errors, email addresses that don't reflect a journal's name, scopes that don't directly align with the recipient's field, and use of incorrect honorifics.

In addition to red flags, it is important for librarians to be vigilant for and caution researchers of clever tactics dishonest journals may employ to fool researchers. With this reasoning, select tactics observed from the author's sample and potential explanations for these tactics are outlined below in Table 2.

**Table 2:** Prevalence and potential explanations of select tactics observed in 60 unsolicited journal emails received between July 20, 2021, and January 31, 2022. Tactics are in the order of most to least prevalent.

Select Tactics Observed	Number of Emails (n=60)
**Name copied from author's past publication in salutation (i.e., “Wilson P.”):** These emails appeared to directly copy the author's name from her past publication (i.e., “Dear Wilson P.”). This could be a tactic to give researchers the impression of the senders being deliberate in their solicitations. As a note, the author's name was publicly accessible via her article in PubMed.	25 (42%)
**Included author's past publication's title:** These emails mentioned the title of the author's past publication in the email. This could be a tactic to give researchers the impression that the sender had read their past publication, and that their future work would be a good fit for the journal. As a note, the title of the author's past publication was publicly accessible via her article in PubMed.	22 (37%)
**Included author's first name in salutation:** These emails included the author's first name in their salutation. This could be a tactic to give researchers the impression of the senders being deliberate in their solicitations. As a note, the author's first name was publicly accessible via her article's abstract in PubMed.	21 (35%)
**Scope matched author's past publication's field because moderately broad:** These emails matched the author's past publication's field of study due to the journals being moderately broad in scope (in this case, clinical trials research, which is somewhat aligned with the author's prior study on clinical trials data sharing). This could be a tactic to give researchers the impression that the sender had familiarized themselves with their work, and that their work would fit the scope of the journal. As a note, the author's past publication's scope could have been extracted from the title of the author's past publication (which included the term “clinical trials”), which could be accessed via PubMed.	15 (25%)
**Included author's past publication's abstract:** These emails included the abstract of the author's past publication in the email. This could be a tactic to give researchers the impression that the sender had read their past publication, and that their future work would be a good fit for the journal. As a note, the abstract of the author's past publication was publicly accessible via her article in PubMed.	12 (20%)
**Claimed to have an impact factor:** Of the journal emails that mentioned impact factors, 3 did not specify the source of the impact factor, 1 cited a fake impact metric, and 3 claimed to have journal impact factors (being a legitimate metric provided by Clarivate's Journal Citation Reports). The author investigated the latter 3 journals in the Journal Citation Reports database [24] and found that none of the journals were present in the database, indicating the journals had likely fabricated their metrics. False or fraudulent impact factors could be a tactic to fool researchers into believing the journal is of high quality.	7 (12%)
**Claimed to be indexed:** Of the emails that mentioned their journal being indexed, only 1 claimed their journal was indexed in databases that are known to employ quality checks prior to indexing, being Scopus and Embase. While the indexing claim for Embase could not be investigated, due to the author not having subscription access to Embase, the author investigated the claim of the journal being included in Scopus and found that the journal's indexing had been discontinued within the past 5 years. 2 journal emails also mentioned having publications indexed in the NLM Catalog (i.e., the physical collection at the National Library of Medicine), insinuating that this was the same as being indexed in Medline (which is not the case!). False indexing claims or boasting of being indexed in databases that have no quality controls, could be tactics to fool researchers into believing the journal is of high quality.	7 (12%)
**Scope directly matched author's field:** These journals directly matched the author's field of study (library science). This could be a tactic to give researchers the impression that the sender had familiarized themselves with their work, and that their work would be a good fit for the journal. As a note, the author's field could have been easily extracted from the name of the journal in which the author had published her past publication (which includes the term “library”), which could be accessed via her article in PubMed.	5 (8%)
**Sender is or is “speaking for” the editor in chief:** These senders claimed to be or claimed to be speaking for the editor in chief of the journal. This could be a tactic to increase the urgency to respond (e.g., receiving a solicitation from a supposed editor in chief could make the researcher feel their manuscript is certain to be included in the journal).	3 (5%)
**Claimed to be following up, though no previous email was received by the author:** These senders claimed they were following up even though they never sent a previous email to the author. This could be attributed to a clerical error on the part of the sender, or it may be a tactic to increase the urgency to respond (e.g., a researcher may be more likely to respond if they believe they may have missed a prior email from the sender).	2 (3%)

Based on the limited observations from Table 2, librarians may consider:

- Warning researchers that potentially predatory solicitors may extract their correct name, field, and past publication information from open databases such as PubMed, and that the inclusion of this information does not necessarily indicate the solicitor is familiar with the researcher or their work.
- Encouraging researchers to investigate any claims of indexing and impact factors, cautioning them of the existence of fake impact factors, and, in the case of indexing, educating them of quality controls (if any!) exercised within a database and their limitations.
- Cautioning researchers to be wary of solicitors claiming to be or to be speaking for the editor in chief, and that mention “following up” on a previous email that was never received by the researcher.

## CONCLUDING REMARKS

Just like predatory journals as a whole, emails from potentially predatory journals have a range of variable characteristics, further complicating their detection. While librarians and researchers shouldn't solely evaluate a journal based on an email, email solicitations are a common tactic employed by predatory journals to attract potential victims. In consequence, librarians need to further investigate and provide support in the identification of potentially predatory journal emails and encourage critical evaluation of the journals that send them. Doing so may play at least a small part in the continued effort to starve these “predators” of their “prey.”

## Data Availability

A deidentified version of the data and data dictionary files associated with this commentary are available in GitHub: https://github.com/weepai/Unsolicited-solicitations-data.
